# Research on the Preparation of Ultramarine Pigments from Palygorskite

**DOI:** 10.3390/molecules30040870

**Published:** 2025-02-14

**Authors:** Min Feng, Qingyun Wang, Xingpeng Wang, Pengwei Mo, Yongchun Tong

**Affiliations:** Key Laboratory of Hexi Corridor Resources Utilization of Gansu, College of Chemistry and Chemical Engineering, Hexi University, Zhangye 734000, China; hxxyfm@126.com (M.F.); w_qingyun@163.com (Q.W.); wangxp@hxu.edu.cn (X.W.);

**Keywords:** palygorskite, solid-phase method, ultramarine, pigment

## Abstract

Ultramarine is a highly favored blue inorganic pigment. It is non-toxic with a deep color and widely used in architecture, plastics, coatings, fine arts and cosmetics. In this study, ultramarine pigment was synthesized using palygorskite, anhydrous sodium carbonate and sulfur as the raw materials through the high-temperature solid-phase method. The incorporation of palygorskite into the synthesis process greatly improves the reaction efficiency and reduces the amount of sulfur. When the mass ratio of palygorskite, anhydrous sodium carbonate and sulfur is 2:6:3, the resulting ultramarine pigment exhibits optimal chrominance. Notably, this sulfur ratio is substantially lower than that used in conventional processes, highlighting the efficiency and potential environmental benefits of this approach. The XRD, FT-IR, UV visible spectroscopy and SEM reveal that the synthetically produced blue pigments possess a sodalite structure, incorporating S_3_^−^ and S_2_^−^ radicals. Stability assessments indicated a marked improvement in the acid resistance of the dark blue pigment upon modification with dodecyltrimethoxysilane, with no notable color degradation observed in either neutral or alkaline conditions. The refined formulation and synthesis process not only optimize the production of ultramarine pigment, but also pave the way for enhanced durability and broader application prospects in various industries.

## 1. Introduction

Ultramarine, a blue inorganic pigment, is favored for its deep color, excellent stability, fading resistance and non-toxicity. It is widely used in construction, plastics, coatings, art, cosmetics and other fields. In the 1820s, J.B. Guimet and C.G. Gmelin invented the synthetic method of artificial ultramarine [1]. In this method, a mixture comprising kaolin, sulfur, sodium carbonate and reducing agents such as charcoal and tar was calcined at high temperatures ranging from 800 °C to 900 °C to produce ultrashine. The industrial-scale production of this pigment was first achieved in Germany in 1834 [2]. In contemporary methods of producing ultramarine, substantial quantities of kaolin and sulfur are utilized as raw materials, with a notably high sulfur content. Typically, the ratio of kaolin to sulfur is 1:1 [3,4,5,6]. The extensive use of sulfur leads to the generation of a large amount of volatile sulfur compounds (mainly SO_2_) during the production process, which is not environmentally friendly.

Given the finite nature of kaolin resources and the increasing emphasis on environmental protection, researchers are actively exploring the use of alternative materials such as zeolite [7,8,9,10,11,12] and fly ash [2,13,14] to substitute for kaolin, while simultaneously aiming to decrease the amount of sulfur utilized in the process to mitigate SO_2_ emissions. Among them, zeolite has achieved relatively good results as a substitute raw material. It is generally accepted that when a sulfur radical precursor is mixed with zeolite and subjected to high-temperature calcination (ranging from 500 °C to 800 °C), the catalytic sites within the zeolite cavities influence the reaction of sulfur compounds. Consequently, the sulfur becomes more difficult to release, leading to a reduction in SO_2_ emissions.

Palygorskite is a mineral material distinguished by its unique crystal structure and surface properties. Its theoretical structural formula is (Al_2_Mg_2_)Si_8_O_20_ (OH)_2_ (H_2_O)_4_·4H_2_O. Its distinctive fibrous or rod-like morphology endows it with a high specific surface area and an abundance of active sites [15,16,17,18]. These characteristics enable palygorskite to effectively adsorb and activate sulfur and related compounds, thereby facilitating chemical reactions and potentially reducing the amount of sulfur required in the process. On the one hand, the distribution and activity of Al^3+^ may favorably interact with other substances, thereby enhancing the synthesis of ultramarine. On the other hand, the silico-oxygen structure of palygorskite offers a more appropriate spatial and electronic environment conducive to the formation of ultramarine, which in turn boosts the synthesis efficiency and enhances the product quality. Additionally, the abundant reserves of palygorskite can readily fulfill the demands of large-scale industrial production.

Although ultramarine pigment is widely recognized as a valuable inorganic pigment, its poor acid resistance somewhat limits its application scope. Consequently, the development of a novel method for synthesizing high-quality, acid-resistant ultramarine pigments is of paramount importance [19,20,21].

This study aims to develop a novel method for synthesizing ultramarine pigment utilizing palygorskite as the primary raw material. The research systematically examines the influence of varying quantities of Na_2_CO_3_ and sulfur (S) on the chromatic properties of the resulting ultramarine pigment. The structural features, morphological characteristics and colorimetric properties of the synthesized ultramarine pigment were thoroughly analyzed using X-ray diffraction (XRD), scanning electron microscopy (SEM) and a colorimeter. Additionally, the acid resistance of the synthetic ultramarine pigment was rigorously investigated to enhance its suitability for practical industrial applications.

## 2. Results and Discussion

### 2.1. Qualitative Analysis of Ultramarine Pigments

**Figure 1:**
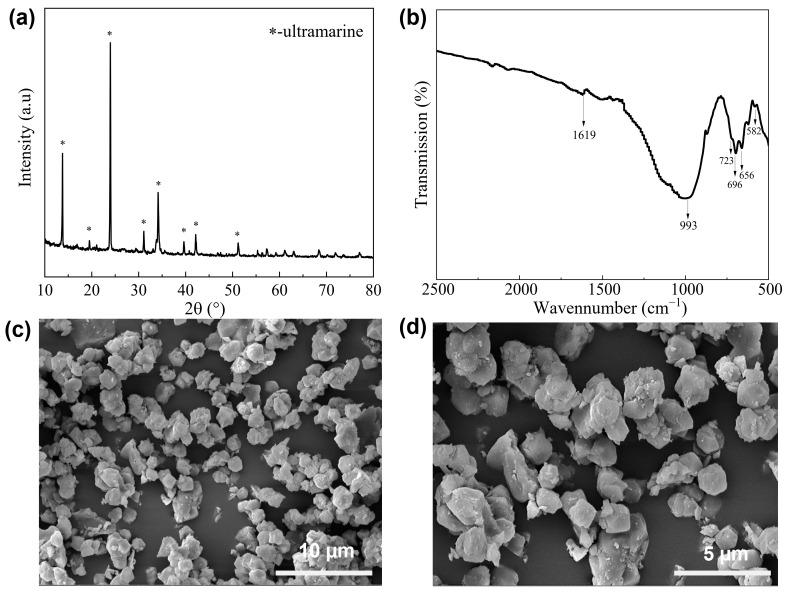
XRD spectra (**a**), infrared spectra (**b**) and electron microscope images (**c**,**d**) of ultramarine pigments at different resolutions.

The XRD pattern of the synthesized ultramarine products is presented in Figure 1a. The diffraction peaks observed at 2θ = 13.70°, 19.40°, 23.80°, 31.00°, 34.16°, 39.54°, 42.18° and 51.14° exhibit excellent correspondence with the reference pattern from the standard sample PDF card No. 77-1702, which represents the cubic crystal blue pigment Na_7_Al_6_Si_6_O_24_S_3_. The XRD pattern demonstrates sharp diffraction peaks with minimal background noise, indicating the successful synthesis of high-purity ultramarine pigment. Fourier-transform infrared (FTIR) spectroscopy was employed to analyze the blue ultramarine samples within the wavenumber range of 500–2500 cm^−1^, as illustrated in Figure 1b. The characteristic vibrational modes at 655 cm^−1^ and 696 cm^−1^ are indicative of the blue ultramarine structure [22]. The absorption peak at 993 cm^−1^, attributed to the Si-O-Al tetrahedral vibration [13], confirms the presence of bridging bonds between silicon and aluminum atoms. The prominent band at 582 cm^−1^ corresponds to the S_3_^−^ vibrational mode, which serves as the primary chromophore in ultramarine pigment [23]. Additionally, the absorption at 1619 cm^−1^ represents the characteristic peak of Na_2_S_2_O_3_. Scanning electron microscopy (SEM) was utilized to examine the morphological characteristics of the ultramarine pigment, as depicted in Figure 1c,d. The micrographs reveal that the blue ultramarine pigment particles predominantly exhibit a rhombic dodecahedral morphology, with minimal agglomeration observed. The particles demonstrate a uniform size distribution, with an average diameter ranging between 2 and 3 μm.

Based on the comprehensive analysis of XRD patterns and infrared spectra, it can be concluded that the synthesized ultramarine pigment possesses structural features characteristic of sodalite. The material can be considered as a sodalite derivative where specific oxygen atoms in the framework have been substituted by sulfur atoms. The unique blue coloration of the pigment arises from the presence of sulfur anions (S_2_^−^, S_3_^−^) that function as chromophores within the cage-like structure formed by the three-dimensional net-work of SiO_4_ and AlO_4_ tetrahedra [24,25]. These chromophoric species are entrapped in the structural cavities or cages of the aluminosilicate framework, imparting the distinctive blue hue to the ultramarine pigment.

### 2.2. The Influence of the Amount of Raw Materials on the Product

#### 2.2.1. The Influence of the Amount of Na_2_CO_3_

**Figure 2:**
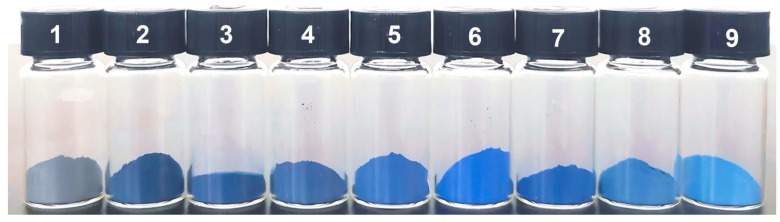
Photographs of ultramarine pigments prepared with different amounts of Na_2_CO_3_ (Samples numbered 1–9 correspond to the products obtained when the amount of Na_2_CO_3_ added are 1.0 g, 1.1 g, 1.2 g, 1.3 g, 1.4 g, 1.5 g, 1.6 g, 1.7 g, and 1.8 g respectively).

The quantity of anhydrous sodium carbonate plays a crucial role in determining the color characteristics and physicochemical properties of ultramarine pigment. This study systematically investigated the influence of the anhydrous sodium carbonate dosage on the chromatic properties and structural formation of ultramarine. Figure 2 presents the photographic documentation of pigments synthesized with varying amounts of anhydrous sodium carbonate under natural illumination conditions. The experimental results demonstrate a distinct color evolution corresponding to the anhydrous sodium carbonate concentration. At a dosage of 1.0 g, the synthesized product exhibits a dark grey coloration. As the anhydrous sodium carbonate content increases, a progressive color transition is observed, evolving from grey–blue to the desired standard blue hue and subsequently transitioning to lighter blue tones. When the quantity of sulfur is set to 0.75 g and the quantity of anhydrous sodium carbonate is 1.5 g, the synthesized ultramarine pigment exhibits optimal color, brightness and saturation.

To quantitatively evaluate the chromatic properties of the synthesized products, the CIELAB color space parameters (L*, a*, and b*) of the ultramarine pigments were systematically measured, as presented in Table 1. The data reveal a distinct correlation between the anhydrous sodium carbonate content and the pigment’s color characteristics. Specifically, the L* value, representing lightness, demonstrates a progressive increase with higher anhydrous sodium carbonate concentrations, indicating a gradual enhancement in the pigment’s brightness. The negative a* values consistently observed across all samples confirm the greenish hue of the synthesized products within the color spectrum. Similarly, the negative b* values substantiate the characteristic blue coloration of the ultramarine pigments. Notably, the blueness parameter (b*) exhibits a non-monotonic relationship with a reagent concentration, initially increasing before decreasing, reaching its maximum value at an anhydrous sodium carbonate dosage of 1.5 g. This optimal concentration point corresponds precisely with the visual observations presented in Figure 2. Furthermore, the color difference metric (∆E*) reaches its minimum value of 1.15 at this optimal concentration, demonstrating remarkable proximity to the standard ultramarine blue reference.

To further investigate the effect of a sodium carbonate addition to the chromophore of ultramarine pigment, the samples were characterized by UV diffuse reflection (UV-VIS-DRS). The structural framework of ultramarine blue pigment consists of a three-dimensional aluminosilicate network, where AlO_4_ and SiO_4_ tetrahedra are interconnected through shared oxygen atoms in a sodalite-type configuration. This colorless framework resembles a cage (β cage) capable of encapsulating small ions and molecules. The synthesis mechanism of ultramarine pigments involves a series of complex chemical processes. Initially, sulfur reacts with sodium carbonate at elevated temperatures to form sodium polysulfide intermediates. Subsequently, the cleavage of S-S bonds generates S_3_^−^ and S_2_^−^ radical anions, which serve as the fundamental chromophores. These chromophore species then migrate into the β-cage structure of the aluminosilicate framework, where they undergo tetrahedral coordination with four Na^+^ cations. This coordination effectively neutralizes the charge imbalance resulting from the isomorphous substitution of Si^4+^ by Al^3+^ in the tetrahedral sites. The stabilization of these polysulfide radical clusters occurs through their encapsulation within the sodium-rich cages, forming distinct NaS_3_ and NaS_2_ complexes that exhibit characteristic blue and yellow coloration, respectively. The final hue of the ultramarine pigment is determined by the relative concentration ratio of these two chromophoric species (S_3_^−^/S_2_^−^) [26,27].

**Figure 3:**
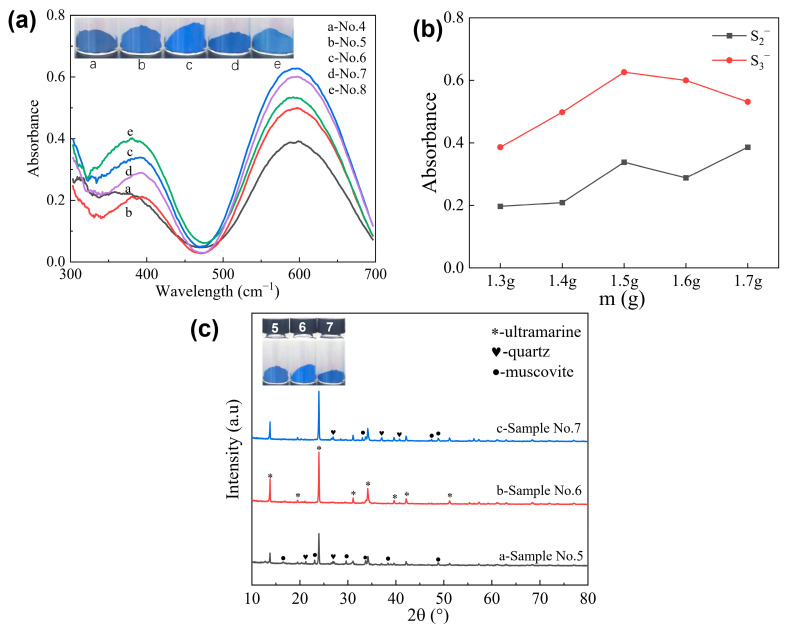
UV profiles of ultramarines prepared with different additions of Na_2_CO_3_ (**a**), the corresponding absorbance intensity of the chromophore (**b**) and XRD patterns of representative products (**c**).

Figure 3a presents the UV-Vis diffuse reflectance spectra of the synthesized samples. The spectral analysis reveals two distinct absorption bands at 395 nm and 595 nm, which are characteristic of S_2_^−^ and S_3_^−^ radical anions, respectively. These bands confirm the formation of yellow and blue chromophores in the pigment, proving effective polysulfide incorporation into the aluminosilicate framework. As can be seen from Figure 3b, the system exhibits a relatively low concentration of both S_3_^−^ and S_2_^−^ chromophores, with a notably higher S_3_^−^/S_2_^−^ ratio. The substantial blue chromophore contributes to the sample’s dark blue appearance. Under conditions of insufficient sodium carbonate, two primary effects are observed: Firstly, the generation of polysulfide becomes inadequate, leading to reduced S_2_^−^ production through direct cracking, with partial conversion to S_3_^−^, thereby intensifying the coloration. Secondly, the relatively excessive sulfur, in the absence of sufficient cations, fails to compensate for the positive charge deficit induced by Al substitution for Si, consequently preventing the stabilization of the chromophore within the beta cage. When 1.5 g of sodium carbonate was added, the S_3_^−^ content reached its maximum level, accompanied by a higher concentration of yellow chromophores. At this stage, the S_3_^−^/S_2_^−^ ratio was measured at 1.85. The sample displayed a vibrant blue color under natural light, demonstrating optimal saturation and closely resembling the hue of commercial ultramarine blue. In contrast, when the sodium carbonate quantity was increased to 1.7 g, the prepared sample exhibited the highest S_2_^−^ content. Previous studies have demonstrated that excessive sodium carbonate facilitates the complete reaction of sulfur [28], leading to the generation of abundant polysulfides. However, the S_2_^−^ produced through cracking cannot be fully oxidized to S_3_^−^ within a short timeframe, resulting in the retention of a significant amount of yellow chromophores in the sample. Consequently, the S_3_^−^/S_2_^−^ ratio decreased to 1.38 and the sample exhibited a light blue coloration.

To further investigate the correlation between the color, purity and crystallinity of the samples, XRD analysis was conducted on samples No. 5, 6 and 7, with the results presented in Figure 3b. Curve b represents the ultramarine sample synthesized with 1.5 g of sodium carbonate. As shown in the figure, this sample exhibits the highest crystallinity, minimal impurity peaks and superior purity. In contrast, curves a and c display more pronounced impurity peaks corresponding to raw materials and intermediate products, which negatively impact the color and brightness of the samples. These observations are consistent with the product’s color under natural light and the UV diffuse reflectance spectroscopy results. The optimal ultramarine pigment is achieved when the anhydrous sodium carbonate content is 1.5 g, corresponding to a mass ratio of palygorskite to anhydrous sodium carbonate of 1:2.8.

#### 2.2.2. The Influence of the Amount of S

**Figure 4:**
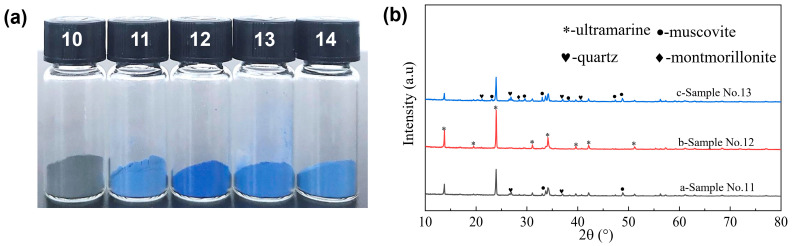
Photographs of ultramarine pigments prepared with different amounts of S (**a**) and XRD patterns of representative products (**b**). Samples numbered 10–14 correspond to the products obtained when the amount of S added are 0.55 g, 0.65 g, 0.75 g, 0.85 g and 0.95 g respectively.

The amount of sulfur plays a crucial role as one of the key factors influencing the coloration of synthetic ultramarine pigments, while also serving as a significant indicator for assessing ecological and environmental safety. Figure 4a presents digital images of ultramarine samples synthesized with varying sulfur concentrations. The results demonstrate that an insufficient sulfur content prevents the formation of ultramarine blue pigment. This phenomenon occurs because the relatively excessive anhydrous sodium carbonate, combined with an inadequate sulfur content, leads to the predominant formation of yellow chromophore S_2_^−^ during the calcination process, while the insufficient generation of blue chromophore S_3_^−^ results in the sample exhibiting a brown coloration. Upon increasing the sulfur dosage from 0.55 to 0.65 g, a noticeable color transition from brown to light blue was observed in the samples. The optimal chromaticity, brightness and saturation of the product were achieved at a sulfur dosage of 0.75 g. A further increment in the sulfur dosage resulted in a gradual lightening of the color. To quantitatively assess the color differences among the samples, the L*, a* and b* values of the ultramarine pigment were measured, with the detailed results presented in Table 2. Notably, Sample No. 10 exhibited a minimal pigment formation, whereas Sample No. 12 demonstrated the highest blue intensity, with an ∆E* value of 0.882. This indicates that the L*, a* and b* values of Sample No. 12 are the closest to those of the standard ultramarine blue pigment, suggesting its superior color fidelity. As illustrated in Figure 4a, it is evident that the ultramarine pigment prepared with a sulfur mass of 0.75 g, corresponding to a palygorskite-to-sulfur mass ratio of 1:1.5, exhibits the most superior color quality.

An X-ray diffraction analysis was conducted on Samples 11, 12 and 13, with the results presented in Figure 4b. The diffraction pattern of Sample No. 12 aligns well with the peak positions of the standard sample PDF card, exhibiting no detectable impurity peaks. In contrast, Sample No. 11 (with a sulfur addition of 6.5 g) displays additional diffraction peaks corresponding to quartz sand and white mica at 2θ = 29.96°, 36.99°, 33.07°, 33.72° and 48.93°. Similarly, Sample No. 13 exhibits more pronounced impurity peaks. Combining the findings from Figure 4a and Table 2, it can be concluded that the optimal synthesis conditions for ultramarine pigment are achieved with an anhydrous sodium carbonate dosage of 1.5 g and a sulfur dosage of 0.75 g. Under these conditions, the S/Na_2_CO_3_ molar ratio is 1:2, which is significantly lower than the 3.5:3 ratio reported in the previous literature. This observed discrepancy can likely be ascribed to the incorporation of palygorskite as a key raw material in the synthesis process. The distinctive fibrous or rod-like morphology of palygorskite confers a high specific surface area and a wealth of active sites [18,29], which significantly enhance the adsorption and activation of sulfur and its associated compounds. Consequently, this promotes the chemical reaction kinetics and elevates the overall reaction efficiency.

### 2.3. Determination and Modification of Acid Resistance of Ultramarine Pigments

#### 2.3.1. Ultramarine Pigment Acid Resistance Test

**Figure 5:**
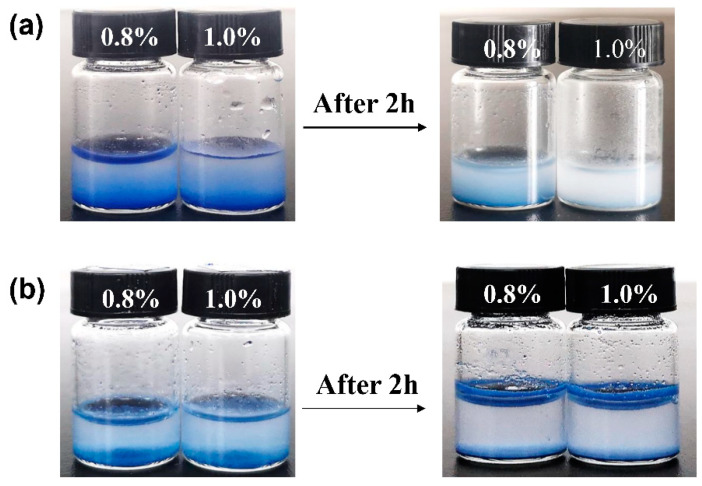
Pictures of unmodified ultramarine (**a**) and modified ultramarine (**b**) after the addition of 0.8% and 1.0% hydrochloric acid for two minutes of oscillation and 2 h of standing time.

The acid resistance of the ultramarine pigments was evaluated using hydrochloric acid solutions with concentrations of 0.8% and 1.0%. As illustrated in Figure 5, the unmodified ultramarine pigment exhibited significant fading and the solution became turbid and milky white. In contrast, the modified ultramarine pigment showed a minimal color change, largely retaining its original blue hue. To further quantify these observations, the chromaticity values were analyzed and the detailed results are presented in Table 3.

Following immersion in hydrochloric acid, the unmodified ultramarine pigment exhibited a significant mass loss and a substantial change in chroma values. In contrast, after modification, both the mass loss and the variation in the chroma values were markedly reduced. This phenomenon can be attributed to the structural characteristics of ultramarine, which consists of a cage-like framework formed by aluminum oxide and silicon oxide tetrahedral units, encapsulating sulfur ions such as S_2_^−^ and S_3_^−^. These sulfur ions are crucial for the blue coloration of ultramarine. When exposed to acid, the hydrogen ions (H^+^) react with the sulfur ions, generating hydrogen sulfide (H_2_S) and elemental sulfur, thereby disrupting the chromophore groups. Additionally, the acidic environment may compromise the crystalline structure of ultramarine, altering its light-scattering and absorption properties. To address these issues, we enhanced the acid resistance of the product through a coating modification process. The acid resistance test results of the modified ultramarine are presented in Figure 5b, demonstrating that the ultramarine retains its characteristic azure color. This improved performance can likely be attributed to the modification of the ultramarine pigment with dodecyltrimethoxysilane. The treatment effectively seals the open-cage structure of the ultramarine particles and forms a hydrophobic, acid-resistant film on their surface. This protective layer significantly enhances the acid resistance of the ultramarine [25].

**Figure 6:**
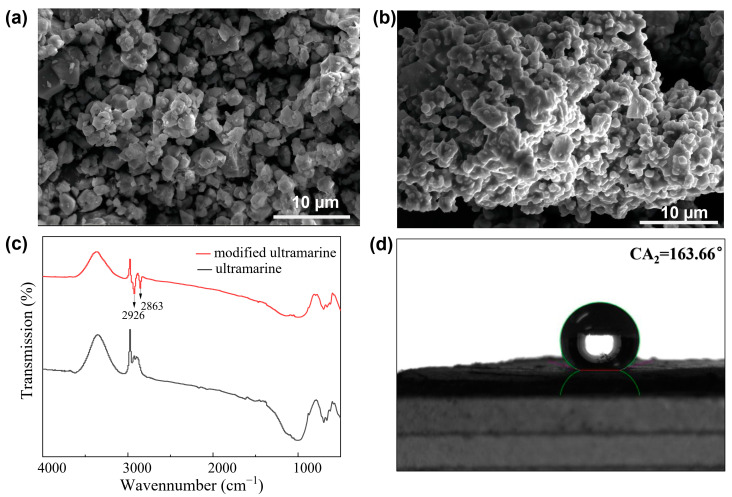
Electron microscopy of ultramarine before and after modification (**a**,**b**), infrared spectra of ultramarine before (black line) and after modification (red line) (**c**), contact angle of modified ultramarine (**d**).

The structural characteristics of the modified ultramarine were systematically characterized. As illustrated in Figure 6a, the pristine ultramarine pigment exhibited a regular morphology with well-defined geometric features prior to the modification and coating processes. Specifically, the particles displayed a characteristic rhombic crystalline structure with excellent structural integrity, featuring sharp vertices and clearly delineated boundaries. Conversely, subsequent to the modification and coating treatment, the morphology of the ultramarine pigment underwent substantial alterations. As depicted in Figure 6b, the crystalline surface is uniformly coated with a protective film primarily composed of a silane coupling agent and its corresponding reaction products. The film, characterized by its consistent thickness and homogeneous distribution, results in a discernible reduction in the sharpness of the crystal morphology. This surface modification significantly enhances the pigment’s dispersibility within matrix materials, improves its interfacial compatibility and augments its durability. Consequently, these modifications substantially broaden the potential applications of ultramarine pigment while simultaneously optimizing its functional performance in various practical implementations.

FT-IR analysis was conducted to characterize the ultramarine pigments in both uncoated and coated states, with the spectral profiles presented in Figure 6c. The comparative analysis revealed distinct spectral modifications following the coating process. Specifically, the coated ultramarine exhibited characteristic absorption bands at 2926 cm^−1^ and 2863 cm^−1^, which are attributed to the antisymmetric and symmetric stretching vibrations of methyl groups, respectively. These vibrational signatures are indicative of the presence of dodecyl chains in the coating layer. Based on these spectroscopic observations, it can be concluded that dodecyltrimethoxysilane has been successfully grafted onto the surface of ultramarine particles through the coating process.

The wettability of modified ultramarine was tested, with the results shown in Figure 6d. After modification, the ultramarine changed from hydrophilic to highly hydrophobic, showing a contact angle of 163.66°. This strong hydrophobicity confirms that the dodecyltrimethoxysilane coating successfully altered the surface energy, enabling new applications in specialized environments.

#### 2.3.2. Chemical Stability Analysis

To assess the chemical stability of the synthesized ultramarine pigments, the modified samples were subjected to immersion tests in various media, including 1% HCl, H_2_SO_4_, HNO_3_, NaOH and H_2_O, representing acidic, alkaline and neutral environments. Following 30 min of agitation, the samples were dried, weighed and analyzed for their L*, a* and b* colorimetric parameters. As summarized in Table 4, the mass losses of modified ultramarine were consistently below 4% across all tested environments. A colorimetric analysis based on ΔE values calculated from L*, a* and b* measurements showed minimal color variations in the range of 0–2.25, demonstrating negligible pigment fading. These results suggest that the modified ultramarine exhibits enhanced chemical stability, with significantly improved resistance to aqueous and acid–base environments compared to conventional ultramarine pigments.

## 3. Experimental

### 3.1. Preparation of Ultramarine Pigments

The first step was purification of palygorskite. A solution was prepared by adding 2.1 g of sodium pyrophosphate to 1400 mL of deionized water in a beaker and stirring for 30 min at 60 °C. Subsequently, 70 g of palygorskite was added and thoroughly stirred. To this mixture, 20 mL of 2 mol/L oxalic acid was introduced and stirred for an additional 5 h. After allowing the mixture to stand for 3 h, the supernatant was separated and centrifuged at 4000 r/min for 10 min. The resulting solid-phase product was washed with deionized water until neutral and then dried at 100 °C in an electric blast drying oven. The subsequent step involved grinding and drying the raw materials. Anhydrous sodium carbonate, quartz sand and sublimed sulfur were finely ground in an agate mortar and then transferred to an electric blast drying oven at 70 °C for 4 h. Purified palygorskite, anhydrous sodium carbonate, quartz sand, sublimed sulfur and rosin were weighed in specific mass ratios, mixed and ground for 20 min. The ground mixture was placed into a ceramic crucible, compacted, covered and calcined in a muffle furnace. The calcination process comprised two stages: The first stage involved heating at 400 °C for 1 h, followed by a gradual increase to 800 °C over 1 h. The second stage involved maintaining the temperature at 800 °C for 2 h. Upon cooling to room temperature, the firing process was completed.

### 3.2. Modification and Acid Resistance Test of Ultramarine Pigments

Modification procedure: The ultramarine pigment was initially treated with a 1% (*w*/*v*) zinc acetate solution through immersion in a temperature-controlled water bath at 70 °C for 30 min. The treated sample was then isolated via vacuum filtration and dried to constant weight. The dried product underwent thermal treatment in a muffle furnace at 200 °C for 4 h under atmospheric conditions. The calcined material was subsequently pulverized using an agate mortar to obtain a homogeneous fine powder. Surface modification was achieved by dispersing the powdered sample in a 3% (*v*/*v*) aqueous solution of dodecyltrimethoxysilane under continuous mechanical stirring. The suspension was then transferred to a forced-air convection oven maintained at 75 °C for 8–12 h to ensure complete solvent evaporation and formation of a uniform surface coating layer.

Acid resistance test: A quantity of 0.15 g of ultramarine was weighed and introduced into separate 2 mL solutions of hydrochloric acid with concentrations of 0.8% and 1.0%, respectively. Each sample was subjected to vigorous agitation for 2 min, followed by a static incubation period of 2 h. Subsequently, the solids were isolated through centrifugation, thoroughly washed to remove any residual acid and dried to a constant weight. The dried solids were then evaluated for their weight retention and chroma values to assess their acid resistance.

### 3.3. Color Tone Evaluation

The colorimetric properties of the synthesized products were quantitatively analyzed using an NR10QC color difference meter (3nh, Shenzhen, China) based on the CIELab (Commission Internationale de l’Éclairage) color space system [30]. This standardized color measurement system provides an objective and reliable approach for color characterization, as it is based on the human visual perception of color through three psychophysical dimensions: lightness, chroma and hue. The color measurements were conducted using the following parameters: L* represents the lightness coordinate ranging from 0 (absolute black) to 100 (pure white); a* denotes the green–red coordinate, where negative values indicate greenness and positive values represent redness; and b* signifies the blue–yellow coordinate, with negative values indicating blueness and positive values representing yellowness. The total color difference (ΔE*) was calculated as the Euclidean distance between the measured values and standard reference values using the following Equation (1), where ΔL*, Δa* and Δb* represent the differences in respective color coordinates between the sample and reference standard.

(1) $$∆E=\sqrt{{\left({∆L}^{*}\right)}^{2}+{\left({∆a}^{*}\right)}^{2}+{\left({∆b}^{*}\right)}^{2}}$$

### 3.4. Characterization

The crystal phase and mineral compositions of the samples were identified using X-ray diffraction (XRD-X’Pert3Powder PANalytical, Almelo, The Netherlands). Phase identification was conducted by leveraging the ICDD (Joint Committee on Powder Diffraction Standards) documentation for inorganic compounds. Fourier-transform infrared spectroscopy (FT-IR) spectra were collected by a Nicolet iS50 spectrometer (Thermo Scientific, Waltham, MA, USA) with a spectral range of 400–4000 cm^−1^ and a resolution of 0.5 cm^−1^ using the KBr technique. FE-SEM measurements were taken with a Quanta 450FEG scanning electron microscope (FEI, Brno, Czech Republic). The ultraviolet diffuse reflectance spectrum was measured using a Lambda 35 ultraviolet spectrophotometer (Perkin Elmer, Shelton, CT, USA).

## 4. Conclusions

Ultramarine pigments were successfully synthesized via a high-temperature solid-phase method employing palygorskite, anhydrous sodium carbonate and sulfur as raw materials. This study revealed that the quantities of anhydrous sodium carbonate and sulfur were critical factors influencing the saturation and structural integrity of the ultramarine pigments. The optimal synthesis ratio was determined to be 2:6:3 for the mass ratio of palygorskite, anhydrous sodium carbonate and sulfur, respectively. Notably, the amount of sulfur required in this synthesis was significantly lower than that reported in previous studies. This reduction not only decreases the cost of raw materials, but also minimizes the environmental impact, offering a more economical and eco-friendly approach to ultramarine pigment synthesis. Furthermore, due to the inherent structural characteristics and generally poor acid resistance of ultramarine, a simple modification using dodecyltrimethoxysilane was found to significantly enhance its stability. This improvement creates favorable conditions for expanding the potential applications of ultramarine pigments.

## Figures and Tables

**Table 1:** Influence of anhydrous sodium carbonate dosage on ultramarine pigment color.

Number	Palygorskite/g	Na_2_CO_3_/g	Rosin/g	Quartz Sand/g	S/g	L*	a*	b*	∆E*
1	0.50	1.0	0.10	0.0717	0.75	≠	≠	≠	≠
2	0.50	1.1	0.10	0.0717	0.75	11.86	−5.09	−18.32	26.22
3	0.50	1.2	0.10	0.0717	0.75	17.67	−5.71	−20.63	20.32
4	0.50	1.3	0.10	0.0717	0.75	24.71	−4.66	−22.29	14.68
5	0.50	1.4	0.10	0.0717	0.75	26.02	−5.00	−28.66	8.81
6	0.50	1.5	0.10	0.0717	0.75	31.93	−6.31	−33.71	1.15
7	0.50	1.6	0.10	0.0717	0.75	33.30	−5.10	−30.87	4.24
8	0.50	1.7	0.10	0.0717	0.75	36.78	−5.64	−25.62	10.29
9	0.50	1.8	0.10	0.0717	0.75	39.91	−9.81	−23.90	13.84

**Table 2:** Effect of S addition on the color of ultramarine pigments.

Number	Palygorskite/g	Na_2_CO_3_/g	Rosin/g	Quartz Sand/g	S/g	L*	a*	b*	∆E*
10	0.50	1.5	0.10	0.0717	0.55	≠	≠	≠	≠
11	0.50	1.5	0.10	0.0717	0.65	42.36	−8.61	−26.92	13.08
12	0.50	1.5	0.10	0.0717	0.75	31.95	−6.27	−33.98	0.882
13	0.50	1.5	0.10	0.0717	0.85	37.28	−5.08	−32.08	5.87
14	0.50	1.5	0.10	0.0717	0.95	40.20	−5.35	−22.34	14.84

**Table 3:** Comparison of acid resistance of ultramarine before and after modification.

HCl Concentration	L*	a*	b*	m_1_ (g)	m_2_ (g)	∆m (%)	ΔE*
0%	31.93	−5.09	−33.71	0.1500	≠	≠	≠
0.8%	26.66	−3.85	−21.07	0.1503	0.1090	27.45	13.75
1.0%	26.35	−3.51	−19.94	0.1495	0.07195	51.87	14.94
0.8%	30.79	−5.01	−32.98	0.1511	0.1422	5.89	1.35
1.0%	29.94	−4.95	−32.89	0.1507	0.1451	3.70	2.57

**Table 4:** Changes in L*, a* and b* values and quality of the ultramarine pigment synthesized by palygorskite after soaking in water and acid and alkali.

1% Acid/Alkali	L*	a*	b*	m_1_ (g)	m_2_ (g)	∆m (%)	ΔE*
Air	31.93	−5.99	−34.71	0.1497	0.1497	0	0.37
H_2_O	32.06	−5.95	−33.98	0.1487	0.1468	1.28	0.89
NaOH	31.61	−5.47	−33.32	0.1481	0.1453	1.86	1.77
HNO_3_	31.98	−5.99	−32.18	0.1490	0.1455	2.34	2.36
H_2_SO_4_	31.64	−5.98	−32.75	0.1495	0.1451	2.97	2.15
HCl	31.22	−5.94	−32.92	0.150	0.1453	3.15	2.25

## Data Availability

The data presented in this study are available in this article.
